# Nurse career mapping: a qualitative case study of a new hospital

**DOI:** 10.1186/s12912-019-0353-z

**Published:** 2019-08-16

**Authors:** Priscylia Maria Sandehang, Rr. Tutik Sri Hariyati, Imami Nur Rachmawati

**Affiliations:** Faculty of Nursing, Universitas Indonesia, Jln. Prof. Dr. Bahder Johan, Kampus UI Depok, Depok, West Java 16424 Indonesia

**Keywords:** Case study, Career mapping, Nurse, Assessment, Competencies, New hospital

## Abstract

**Background:**

Career mapping is an effective strategy for providing nurses with a clear direction and a realistic time scale for achieving their career goals. The purpose of this research is to investigate career mapping for nurses at a new hospital in Jakarta.

**Method:**

The study design is qualitative and implements a focussed case study approach. Data were collected from focus group discussions (FGDs) with two groups: one group consisted of eight staff nurses and the other consisted of six nurse managers. An inductive content analysis of all transcripts from the FGDs and of field notes was conducted independently.

**Result:**

This research produced seven themes that together portray the entire career mapping process. In Hospital X, the career map for a particular nurse is based on the level of formal education, the length and nature of their work experience and a competency assessment. A self-assessment process and considerations related to competence in all aspects of nursing have been included in the process for nurses at Hospital X. The idea that nurses should be positioned in a working environment that matches their level of competency is a fundamental principle for nursing managers.

**Conclusion:**

As a new hospital, Hospital X has implemented nurse career mapping and striven for accreditation. Career developments not only become the responsibility of an organisation but also the responsibility of individuals to develop themselves and their careers.

**Electronic supplementary material:**

The online version of this article (10.1186/s12912-019-0353-z) contains supplementary material, which is available to authorized users.

## Background

Nurses are the most prodigious human resource in healthcare. Nurses work in shifts, interacting with patients, doctors, colleagues and other health experts while enduring high work demands, a rotational work schedule and daily emotional impositions that must be dealt with to avoid boredom and fatigue [1]. Many nurses who possess the capability to grow and develop at a hospital become frustrated, lack motivation and eventually resign. Therefore, it takes travail to retain and develop nursing skills and careers, one of them with the career stages programme [2]. Retention programmes include career stages and nurse rewards [3].

A professional career stage is a system to improve performance and professionalism, field-based competency [4], work satisfaction and resolve differences in the amount of remuneration workers believe they deserve [5]. The success of a career depends on the nurse’s participation in clinical decision making, the ability to define short-term and long-term goals and the ability to manage his or her nursing career proactively [6]. Nurses must realise that they need to improve their competence, while their leaders need to accept their responsibility to facilitate this process and support the nurses with developmental policies [7]. Research in Colorado (*n* = 68) found that many registered nurses decline to participate in career stages due to a lack of realisations toward career stages and the application process [8]. Nurses’ participation in the career stage is also instigated by intrinsic motivation. Career stages that are contrived to appreciate clinical advantages and nurses’ competencies require an active role to achieve the targeted career level [9].

Mapping is a process in ordaining nurses’ levels based on prerequisites set by the hospital. The world has recognised career mapping patterns since the 1970s to guarantee improvement in nurses’ competencies and the quality of services. In Indonesia, the career stage was only implemented in 2005. Career stage figures used by the hospitals in Indonesia had to be modified to adapt to the hospital conditions in 2006. Proper career stage system implementation requires a competency evaluation supplemented by a self-assessment. The results of a literature review and discussions with practitioners in the hospital showed that there are some captivating elements regarding career stage implementations in Indonesia.

In fact, Indonesia has had a legal basis that enforces career stage implementation procedures. However, the enactment of career stage implementation appears to be solely to achieve accreditation. In addition, career stage implementation in some hospitals has experienced obstacles and various career stages models that will be performed. This has been the case since the earliest stage, and the mapping process still creates misunderstandings and variations in career stage implementation as well as protests or conflicts during mapping.

There are almost no meaningful adjustments when a nurse is positioned at a certain career level. Sometimes, nurses’ competencies and their duties do not match their career level. Hospital X is a new hospital that serves as an interesting case study. Of the various methods presented, the researcher will investigate nurse career mapping in Hospital X.

## Methods

### Participants, data collection and design

The research was conducted in a new general hospital established in 2015 in Jakarta with 455 beds. The capacity includes: a VIP (Very Important Patient) unit with six beds, an inpatient unit with 359 beds (divided into Class I with eight beds, Class II with 39 beds and Class III with 312 beds), an HCU (High Care Unit) with 18 beds, an ICU (Intensive Care Unit) with 15 beds, an ICCU (Intensive Cardiology Care Unit) with seven beds, an NICU (Neonatal Intensive Care Unit) with 13 beds and a PICU (Paediatric Care Unit) with seven beds. The nursing staff consists of 376 people, of which 225 have attained a diploma, one has a Bachelor of Nursing and 150 are registered nurses. The staff only comprise 68% of the hospital standard of 468 nurses. The nurses’ workloads are excessive, with the ratio of nurses to patients in one service at 1:10–12. The bed occupancy ratio is as high as 90%.

This research used an intrinsic case study approach that aims to establish a comprehensive understanding of nurse career mapping. A purposive sampling technique was applied to acquire 14 participants who were divided into two groups: eight nurses and six nurse managers. The participation criteria required various nursing education levels. The first group was comprised of staff nurses and the team’s captain; the second group included the nurses’ managers from the lower, middle and top levels and diploma and registered nurses with work experience ranging from less than one year up to 16 years. All participants who agreed to participate in this study signed the informed consent form to participate and consent to publicise the research result. The qualitative research instrument is self-researcher [10]. The researcher was a facilitator, who was helped by a documenting assistant. Aides included structured question guidelines, field notes, stationeries, two voice recorders with 4GB memory capacity that were positioned throughout the hospital during the FGD implementations and a handphone as a backup should there be any technical issue with the recorders.

### Data analysis

An inductive content analysis was used and corroborated with the research team. Audio data were transcribed and supplemented by field notes. Transcripts were read twice to catch any writing errors and to confirm the participants’ statements; the participants were involved in validating the transcripts. Then, the data were organised, extracted to find meaningful statements and parsed to formulate meanings. The data were then categorised to be formulated into themes. Gradually, they were condensed into codes and integrated in a complete breakdown of the researched cases. Ultimately, the data were presented as research results in the forms of figures, tables and discussion materials.

### Data validity

Data validity tests were performed in several ways. First, participants checked the conformity of their statements with the transcripts and field notes. Second, proof of the data collecting process until whole data can be outlined. Research evidence was conserved in audio recordings and field notes. Third, transcript data, field notes and data analysis results were discussed with an expert on qualitative research. The research results were controlled by receiving validation from the participants and comparing the research results with related literature. Fourth, the application of the principle of data transparency was complimented by submitting clear reports that were detailed, systematic and trustworthy so that they could become a reference for readers.

## Results

The participants’ characteristics in this research are described in Tables 1 and 2. Pseudonyms were used for every participant. The flow and requirement of nurses’ career stages are included in a file attachment in Figs. 1 and 2 (see Additional file 1).

**Table 1:** Participants in FGD Group 1 / Staff Level

No	Pseudonym	Sex	Position	Education
1.	Lasmi	Female	Primary Nurse at Maternity Unit	Diploma Nurse
2.	Titis	Female	Primary Nurse at Internist Unit	Registered Nurse
3.	Dewi	Female	Associate Nurse at Maternity Unit	Diploma Nurse
4.	Puja	Female	Associate Nurse at Maternity Unit	Registered Nurse
5.	Ayu	Female	Associate Nurse at Maternity Unit	Diploma Nurse
6.	Maya	Female	Associate Nurse at Internist Unit	Registered Nurse
7.	Ningsih	Female	Associate Nurse at Internist Unit	Registered Nurse
8.	Wiwin	Female	Associate Nurse at Internist Unit	Diploma Nurse

**Table 2:** Participants in FGD Group 2 / Managerial level

No	Pseudonym	Sex	Position	Education
1.	Nita	Female	Director of Nursing (Top Nurse Manager)	Registered Nurse
2.	Dian	Female	Head Nurse	Diploma Nurse
3.	Bima	Male	Head Nurse	Registered Nurse
4.	Siti	Female	Head Nurse	Registered Nurse
5.	Fika	Female	Head Nurse	Diploma Nurse
6.	Bilqis	Female	Head Nurse	Registered Nurse

As this is an interpretivist study, the researcher included her interpretation while translating the transcript into English to avoid the possibility of content loss in the translation process. This research effectuates seven themes, and examples from the participants are included below.

### Career stage mapping requirements based on education level, work experience, work duration and competencies

Participants discussed the qualifications or considerations needed to map nurses’ career stages, which include education level, work experience, work duration and competency, as elaborated in the statements below.

*“In my opinion, nursing career mapping can be determined by the educational levels.” (Ayu #FGD1).*

*“Career stage mapping starts with how the nurses will be positioned, based on their employment duration.” (Lasmi #FGD1).*

*“So, nurses need to do self-assessment to know their qualities and abilities as a nurse.” (Dewi #FGD1).*

*“Every nurse must have career stage. Time passes, they must improve their competencies by reaching higher education level or taking short courses for certain skill in the nursing profession. So, the nurses will be promoted faster or will be given the chance to progress in their career level as soon as possible.” (Lasmi #FGD1).*

### Career stage processing-based mapping model modification

The career stage mapping model in Hospital X is initiated by completing administration files, preparations, assessments, a results appeal and evaluation-monitoring. Participant statements that represent this theme are as follows.

*“As the first process of the career stage, we prepare the nurses’ data. From the first time they work in our hospital, we ask them to provide the birth certification, certificates of degree, letter of work experience followed by previous work experience, or any course certificate that is in line with nursing. They have to compile it on a flash disk and upload it to the HRD’s system.” (Siti - FGD2).*

*“We have competency assessments arranged by assessor, skill examination, self-evaluation and peer review. But we are not strict. Assessees can know who their assessor will be and which unit will be used as an assessment spot. We modify this based on the hospital’s conditions. I mean that the assesee who failed can complain if they think that they are already good enough to pass the examination. After that, the assessor will reanalyse their complaint.*” *(Siti - FGD2).*

*“We did the competency assessment, peer assessment and self-assessment as proof and validation of the nurses’ competencies” (Nita - FGD2).*

### Whole elements of nursing in the process of career stage mapping

Hospital X involves all elements of nursing in career stage mapping, whether they are associate nurses, nurses’ managers or nurses who have participated in career stage mapping training. This theme is supported by the statements below.

*“We also involve our colleagues, who were recruited as assessors in their previous workplace. We had extra workdays, and they participated, even though they have the right to a free day. We work together in the whole process of assessment and build a strong team for it.” (Fika - FGD2).*

*“We involved head nurses and primary nurses in every unit to carry out socialisation regarding the process of nursing career mapping. As a result, they understood and helped the nursing career mapping team in this process…” (Nita - FGD2).*

### Obstacles faced by the managerial level and nurses in implementing career stage mapping

Some obstacles faced by nurse managers include incomplete administration papers, time limitations, a lack of motivation from nurses and limited support from hospital leaders. Some obstacles faced by implementing nurses include complex question formats, time limitations and difficulties with self-evaluation. Statements that represent this theme are provided below.

*“The other obstacles come from completing the documents. The majority of nurses do not complete all of their documents: They do not have the work experience letter, degree certificate, or registered nurse’s certificate.” (Bilqis - FGD2).*

*“I think the obstacles are a lack of time and low numbers of human resources. Even though we are the assessors, we have to back up nursing services in certain units.” (Nita - FGD2).*

*“There is no intrinsic motivation yet from the majority of nurses for developing themselves. We understand this condition, because it is still a new hospital and a new nursing workforce. They need to suit all programmes. But hopefully in the near future they will have the self-realisation and high intrinsic motivation to arrange their careers.” (Fika - FGD2).*

*“Generally, our hospital management’s team think that the mapping document is just a piece of paper and that it is useless. They do not yet realise that it is important in helping nurses to develop their skills and knowledge, which impact on the quality of nursing-care delivery” (Siti - FGD2).*

*“Since there are too many forms needed to be completed, we felt it was a little bit difficult to do. Actually the competency-evaluation form had been completed, but other forms are confusing and time consuming,” (Maya - FGD 1).*

*“As you know, we fill out the self-evaluation forms according to our opinions, so it is more subjective. I mean, what I think about something is like that, but according to other people, it is not like that. I think the other obstacle is that judgement tends to be subjective.” (Puja #FGD2).*

### Creative solutions for overcoming career stage mapping obstacles

Nursing elements in Hospital X possess some steps to overcome these obstacles, including: special tricks in outpatient rooms, coordinating administrative papers collectively and promoting internal training. This theme is supported by the following statements.

*“We have tips that have been given regularly to prepare the nurses in our ward for the competencies assessment. Actually, they only have to read again or recall the operational procedural standard (OPS) of every nursing intervention or collaborative intervention. Nurses rarely read it, so they forget about OPS when they are assessed. So, in this short amount of time, we usually spend ten minutes reading an OPS after handover. Nurses said that in this way they are able to remember it or discuss it with their peer group in terms of the SOP. At the end, they will be more confident and ready to face the assessment day” (Dian - FGD2).*

*“When we find competency gaps, we fix them internally. We are very glad that many of our friends are intelligent, so we can better discuss it. We also conduct short training and discussion sessions or share our reflections in journals and case studies twice a week” (Dian - FGD2).*

### Response evaluation and career stage mapping benefits

The results and benefits of career stage mapping were evaluated, and the responses from nurses and other health workers were diverse. Significant statements that supported this theme include:

*“We get good responses from the doctors and midwives. They are very supportive of implementing the nursing career mapping programme. The doctors and midwives also think that if the nurses are competent in giving nursing care, they will be happier, as will qualified nurses, who will work together to provide the best healthcare services.” (Ayu - FGD1).*

*“Nurses respond to this programme in different ways. Many of them are afraid because of the decline in their nursing career level. If they are evaluated, then the result will show that they are incompetent on a certain level. They know that this will mean a decrease in their career level. This will have a bad effect on their remuneration. That is the point that scares them about the programme of a mapped nursing career.” (Fika - FGD2).*

*“In our ward, we motivate each other. We explain that actually, we will only be observed in terms of our nursing intervention, so do not be afraid. We help each other to prepare for the assessment.” (Dian - FGD2).*

Benefits of career stage mapping include favour in implementing duties, achievements based on competencies, providing clinical authorities, training, financial incentives, self-motivated development, inter-professional collaborations and conducive organisational environments. This theme is supported by the statements below.

*“If we see from the mapping that is very good because it prioritises nursing duties… The mapping used is a basic human need that encourages the nurses about their main duties in care, not just as medical assistants.” (Lasmi - FGD1).*

*“The one type of reward that we have given is to send some nurses to short training session for improving their professional nursing skill.” (Nita - FGD2).*

*“One of the targets in nursing management division is that all of nurses in this hospital will have their career level so that they will get the remuneration based on their career level and performance” (Nita - FGD2).*

*“With the existence of career mapping programmes, it will help nurses to carry out their duties and responsibilities. Nurses are able to perform in accordance with their competences. There are responsibilities and accountabilities in every action performed by nurses. Besides this, healthcare staff become more comfortable working with nurses and opening up discussions about patient health.” (Wiwin - FGD 1).*

*“Through this process of nursing career mapping, our togetherness is guaranteed, more loyal and support each other more.” (Nita - FGD2).*

### Follow-up plan of the career level mapping programme based on nursing staff development

Nurse managers have developed a follow-up plan to the career stage mapping programme by trying to maintain the image of the hospital, improving human resources in nursing education, evaluation and prolonged monitoring, establishing a mentorship programme, satisfying the fundamentals of formal and non-formal education, collaborating with educational institutions, providing positive support and simplifying the assessment format. Some statements that support this theme follow.

*“We always help our friends to understand that we all work in the public hospital. As we know, many public hospitals receive complaints from patients and society. The health workers in hospital, especially nurses, are still stigmatised for being too serious or tense. Now, we want to change that image by providing the best nursing services for society.” (Nita - FGD2).*

*“After career mapping is performed, we will evaluate its implementation. Hopefully there will be more feedback for the career mapping programmes so that they will become better.” (Lasmi - FGD1).*

*“We hope to continue this programme for maintaining nurses’ competencies. We agree and support the evaluation of nurse competencies that is done by evaluating a nurse’s ability to fulfil the basic needs of patients” (Puja - FGD1).*

*“Through this process, we can also see that nurses share their knowledge and skill. For example, if a nurse at level 1 do not know about any skill then nurses level 2 will guide those nurses at level 1.” (Titis - FGD1).*

*“In the near future, there will hopefully be an opportunity for nurses to continue their formal higher education as a follow up to the nurse career mapping programme.” (Dewi - FGD1).*

*“For the next three years, we should collaborate with some education institutions that deliver the practice of nursing clinics in our hospital. In 2018, we are expecting those institutions to admit some of our nurses to their institutions for continuing formal education. So there will be mutual benefits.” (Nita - FGD2).*

*“We hope that no one will play favourite from hospital management in choosing which nurse should partake in continuing education or formal training. My hope is that the management division will support every nurse in develop their competencies.” (Fika - FGD 2).*

## Discussion

### The requirements and career stage mapping model

Career stage mapping in Hospital X is categorised by education, experience, work duration and competency evaluation. This career staging has its own unique ways of assessing personal capabilities and self-assessments. Benchmarking results can be combined into personal communications with the nurses’ managers, who apply career stages in some Indonesian hospitals, suggesting that there are many duties to be fulfilled [11]. The career stage has been implemented in some hospitals but is sometimes insufficient for recruitment implementation, rotations, prolonged professional improvement and promotions, which become inseparable components of career stages [12, 13].

National hospitals still have nurses with only high school educations, and career stage regulations have been considered. This will become the role model for other hospitals in Indonesian provinces. A profession should determine the minimum qualification of a profession’s level and no vocations below it [12]. As a recently established profession that has only recently been protected under law in Indonesia through the enactment of Nursing Law no. 38 in 2014, nursing is still very much in need of benchmarking. It needs to learn from other countries that have established regulations for ensuring the quality of their nursing. This quality needs to be established in terms of the nursing services provided and regulations that protect nursing staff; it must also ensure that an adequate nursing education system is established. However, better resources are required to establish a nursing system in Indonesia and strengthen the nursing profession. Benchmarking results in Thailand indicated that no nurses there have a vocational education [14]. This consequence should be addressed by the recognition of a profession [15].

Nurses’ career paths in Japan, Taiwan and Thailand begin with basic nursing education, followed by license issuance with certain qualifications. Specialist nurse development must focus attention on the qualitative aspects of professional experience in services and support the right individual for human resource improvement [16]. Nursing careers in England since 2006 have been directed into five career pathways: society and family heath, emergency and critical care, first contacts, access and emergency care, long-term care support and mental and psychosocial care [11]. Nurses need to improve their competencies [17]; thus self-evaluation becomes one of the strategies to build a nursing practice and proof-based career stage [18].

Competencies are interconnected to education and formal and informal training. Nurses’ competencies need to be clearly understood based on the stages [19]. Higher education is conducted for professional improvement and quality nursing care. Human resource management executes recruitment and retention of skilled employees so that hospitals can provide quality services [20]. Organisations can achieve competitive superiority through their talented, skilled and experienced staffs. One way to ensure that nurses achieve maximum performance is through career stages [21].

Career mapping requires guidelines that cover the implementation of career stage development, competencies that need to be fulfilled and other options for career development [17]. Self-evaluation and assessment verification can become one of the strategies to establish a nursing practice culture and career stage systems that adopt proof-based nursing competencies [18]. Competency assessments at the new hospital are a good starting point for helping management determine the nurses’ capabilities and provide quality nursing care. Early mapping competency assessments are unequivocal because the organisational culture is easily shaped and nurses will gain increased motivation from improvement programmes. Many hospitals have operated for years but still have difficulties developing a competency assessment due to the static attitudes of nurses who are apathetic toward many development programmes [22].

### Career stage mapping increases service quality and substantiates nursing professions

Workers who tend to improve themselves are emboldened by the highest basic human necessity, which is the need for self-actualisation [23]. Nurses feel proud of their career level. The career stage effectively supports professional career development, keeping quality nurses working at a hospital, and also serves as a self-actualising vessel [24]. This requires support from leaders and colleagues. The career stage sometimes becomes just a concept without further action. Nurses in Indonesia tend to think of career stage implementation as the promise of additional financial rewards, when in reality, career stages are implemented to guarantee service quality and nurses’ competencies, so they must be interpreted as a medium for motivating professional development [25]. Career stages, according to nurses, are their competency responsibilities.

Career stages can increase work satisfaction, quality care provisions, safety and are cost-effective for patients [26]. Nurses need to understand that the purpose of career stages is to improve service quality and substantiate the nursing profession. Career stages also impact financial rewards and clinical authority, but nurses are also required to exhibit exemplary working conditions [27]. Career stages provide clinical authority, in which nurses who are considered competent will do the work determined by a formal review process by a group of people including professional colleagues and specialists based on their qualifications, education, skills and experience [23]. This process includes the determination of clinical competence [28]. Nurses’ competencies must include a core competence that relates to their specialties.

Nurse managers stated that in Hospital X, the sense of urgency to implement career stages was to gain accreditation. These concepts are not precise, so it needs to be emphasised that four standards exist in accreditation evaluation, but career stages are not a prerequisite. The goal of accreditation is increased quality of service, not achievement of the bare minimum. Career stages must be viewed as a medium for achieving accreditation but not as the final destination.

### Nursing synergy elements are involved in career stage mapping

Career mapping involves lower-line to top-line nurses. Human resources are needed by nurse managers to clarify the development process after nurses achieve the roles to plan for education continuance [8]. Nurses’ elements in Hospital X indicate the totality in the process of career stage mapping until the preparation for accreditation. Organisations need leaders’ sustenance for the success of the programme. Top management needs to be active from the beginning of mapping until the nurses’ careers are determined [7]. Competency reviews and goal preparation help employees identify competencies that must be reached [29]. Organisations need to organise work limitations and management performance through competency arrangements and reward mechanisms that touch base with professional aspects to establish achievement motivations. Organisations motivate employees by providing opportunities to express ideas, as making decisions is an important factor for increasing performance [30].

Nurses are individually responsible for improving themselves [23]. Career stages are simply a medium and cannot be relied on to promise career developments. Nurses must conscientiously and purposely plan out their careers in a continuous process [29]. The responsibility of an individual to strategise career development is a difficult concept for Indonesian nurses. It is difficult to plan for self-improvement individually, such as through training programmes, because the people who are assigned to join the training are the same people, and many are managerial nurses. Development programmes are in the leaders’ dominion, and staff are not capable of voicing their development aspirations. Transcendental achievements, overlapping all things that seem to be material advantages, need to become individual motives for self-improvement.

### Obstacles of the career stage mapping process and their solutions

Obstacles of implementing career stages are experienced by nurses as well as nurse managers. Nurses feel a lack of support and see it as a problem in the institution’s system as well as direct self-prevention [31]. Some nurses cited an excess of documents to be completed and the difficulties, complications and time consumption associated with applications as obstacles [32]; these obstacles also occur in other countries. Another obstacle is that nurse managers feel the inadequacy of the hospital’s support for nursing. Leaders’ patronage becomes a serious concern because health workers need to feel appreciated to be motivated to work [33]. Nurse managers are blocked by a lack of financial support and understanding about professional development and do not have power at the middle management level to enact professional improvements. Nurses are not considered important contributions to human resources in the planning of professional development [1].

Other obstacles include obscure duties and nursing committee roles. Nurses in the committee include the room master and team leaders, who are responsible for 4–6 patients daily and who execute the committee’s assignments. Structural positions cannot be the reason for swaying the main tasks to provide nursing care. Suggestions include limiting clinical duties and providing manageable nurse-to-patient ratios that are suitable so as not to overload the nurses and allow them to execute their duties optimally, whether in functional or structural fields.

In addition, there are misconceptions about the assessor concept at Hospital X. Nurses who participate in assessor’s training return to the hospital to execute it, and the room master and one team leader from every team in each room must be an assessor. Assessors do not perform nursing duties to reach the target of assessed nurses and believe they are exempt from delivering patient services. Assessors should be nurses who possess credibility and adequate clinical skills to master competencies, especially if the assessed area overlaps with the competencies in a special nursing unit such as the ICU or HCU.

### Career stage mapping programme evaluation

Career stages provide clarification about financial rewards [34]. Financial benefit is known as remuneration, which increases motivation to work [35]. The nursing manager’s role is to determine nurses’ remunerations based on performance evaluations [36]. Remuneration implementations are based on the workload, nursing care provided to patients, education level, work duration, responsibilities, attendance and position of each nurse [37].

Competency-based career mapping supports health workers’ collaborations and can increase patients’ safety and ease of access, coordinate effective health services, optimise the health of health service providers, develop mutual respect, share knowledge and skills and escalate roles and communications [38]. Nurses in Hospital X hope for the mentorship process to lay the foundation for professional development. Mentorship promotes self-efficacy by escalating confidence, competencies and pride. Self-efficacy has a positive influence on involvement in professional development and career progress [39, 40].

## Conclusion

Hospital X is a newly opened hospital that has implemented nurse career mapping and has actively worked to achieve accreditation. A new hospital employing nurses with a heavy workload is at an advantage in promoting development programmes. Hospitals need to prepare their nursing workforce through clear planning. This needs to become an on-going process that is used to align the priorities of the organisation with those of its workforce to ensure it can meet its service requirements and the behaviours expected of the organisation. An assessment of competencies at the start of career stage mapping can direct nurses to master core competencies. This is a basic requirement that can be a significant advantage to the hospital. Inaccurate work placements, where nurses lack the necessary qualifications or competencies, can cause nurses to under-perform and fail to provide optimal services. Not only is career development the responsibility of an organisation, it is also the responsibility of each nurse to improve his/her own career without depending on management. Career mapping needs to be understood as an effort for professional development as well as adequate nursing care, not just for fulfilling accreditation requirements.

## Additional file

Additional file 1: contains Figs. 1 and 2, which explain the flow of career mapping and the requirements of nurses’ career levels in Hospital X. (PDF 474 kb)

## Data Availability

Not applicable.
