# Supplementary material for: Nurse career mapping: a qualitative case study of a new hospital
Source: BMC Nurs. 2019 Aug 16;18(Suppl 1):31. doi: 10.1186/s12912-019-0353-z (PMC6696660; doi:10.1186/s12912-019-0353-z)
Supplement: Supplementary file 1 — contains Figs. 1 and 2, which explain the flow of career mapping and the requirements of nurses’ career levels in Hospital X. (PDF 474 kb) [file 12912_2019_353_MOESM1_ESM.pdf]

Figure 1. Career Stage Mapping Flow in Hospital X

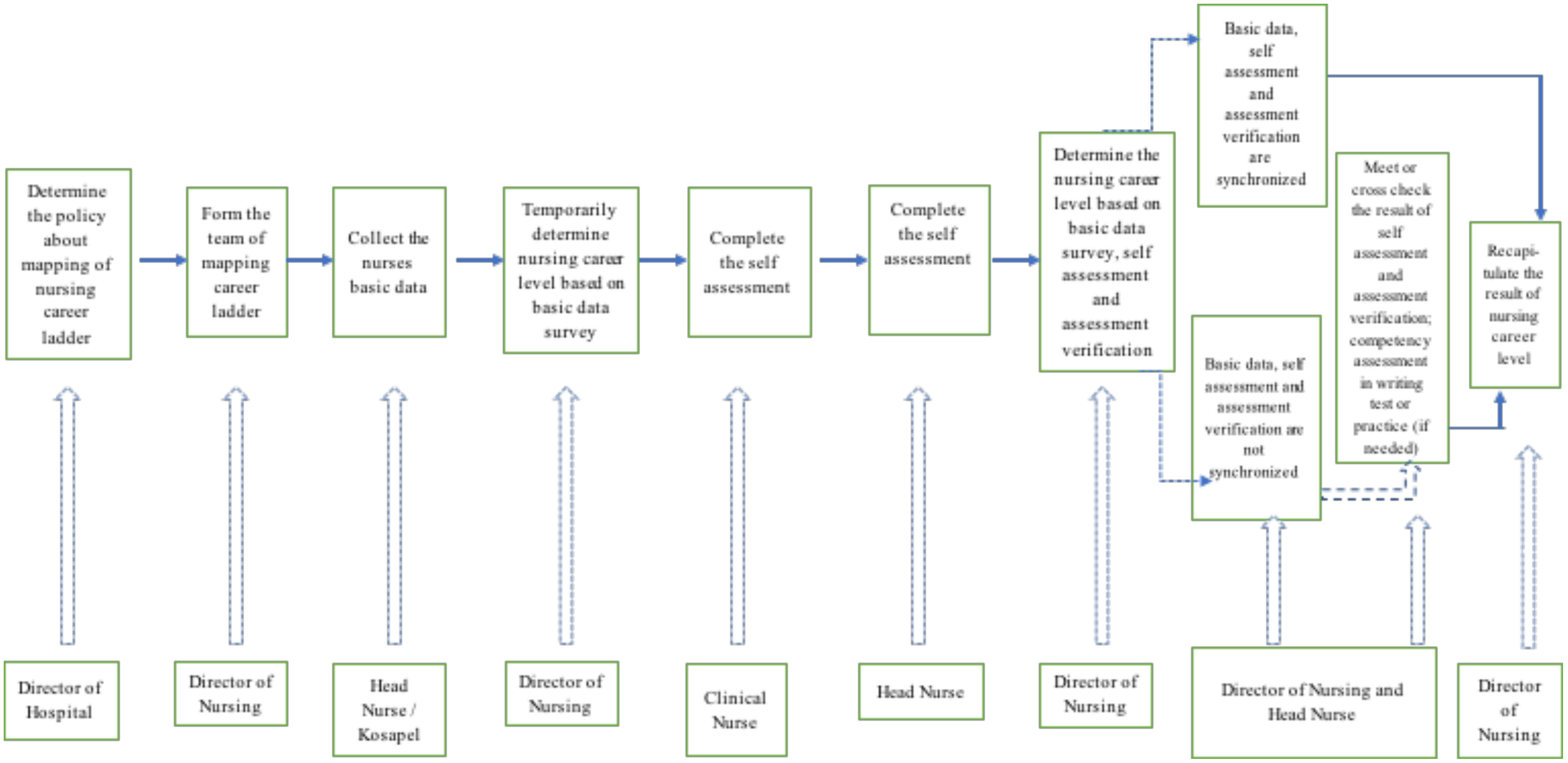

Figure 2. Nurses' Career Level in Hospital X

PK IIIC	• Specialist Nurse + min. 6 years work experience<br>• Ns + min. 15 years work experience<br>• Nurse Diploma + min. 17 years work experience	PK IIIC: 90% of Total Competencies*	391 Total Competencies*
PK IIIB	• Specialist Nurse + min. 3 years work experience<br>• Ns + min. 12 years work experience<br>• Nurse Diploma + min. 14 years work experience	PK IIIB: 70% of Total Competencies*	
PK IIIA	• Specialist Ns + 0 year experience<br>• Ns + min. 9 years work experience<br>• Nurse Diploma + min. 11 years work experience	PK IIIA: 50% of Total Competencies*	
PK IIB	• Ns + min. 6 years work experience<br>• Nurse Diploma + min. 8 years work experience	PK IIB: 80% of Total Competencies*	292 Total Competencies *
PK IIA	• Ns + min. 3 years work experience<br>• Nurse Diploma + min. 5 years experience	PK IIA: 60% of Total Competencies*	
PK IB	• Ns + 0 year experience min.<br>• Nurse Diploma+ min. 2 years experience	PK IB: 80% of Total Competencies*	162 Total Competencies*
PK IA	• Nurse Diploma + 0 years experience	PK IA: 60% of Total Competencies*	

Notes:
* = Amendable amount of competencies based on ward conditions, if it has not been done or irrelevant based on ward conditions, then the competencies uncountable and given a code (T)
